# New Performance of the BJCVS

**DOI:** 10.5935/1678-9741.20160083

**Published:** 2016

**Authors:** Domingo M. Braile

**Affiliations:** 1 Editor-in-Chief - BJCVS

The Brazilian Journal of Cardiovascular Surgery (BJCVS) has been going through a restructuring phase and has also conquered a large number of legacies in the last two months. We had the opportunity to notice the interest of the Associate Editors, Junior Editor, Reviewers, Authors and Contributors, thus, helping our journal to have a future of progressive steps, increasing the frequency and regularity of our publications.

## ASSOCIATE EDITORS

The BJCVS currently has 13 volunteer Associate Editors who are striving to fit the requirements of the new submission system in addition to capturing articles in the respective areas: Advances in Cardiovascular Surgery, Diseases of the Aorta, Basic Research, Cardiovascular Surgery, Cardiovascular Rehabilitation, Continuous Medical Education (CME), Congenital Diseases, Coronary Surgery, Transcatheter Aortic Valve Implantation (TAVI) and Valvar Surgery.

## SUBMISSION SYSTEM

The submission system has already received 50 manuscripts for evaluation. The barriers are being broken, and with each obstacle the BJCVS team develops a commendable advisory work so that all doubts are quickly cleared up.

We are involved in a rigorous process for meeting deadlines. Reviewers who do not meet deadlines are replaced, resulting in the automatic generation of negative punctuation on ScholarOne evaluation system. Likewise, Associate Editors are also evaluated according to the fulfillment of deadlines and quality of opinions.

We are very excited about this systematics, because Authors who do not submit the manuscript correction within the determined period, have the manuscripts invalidated, and must submit it as a new manuscript, losing its position in publication queue.

## MANUSCRIPTS IN ENGLISH

Spelling and Grammar have strongly been questioned by the Reviewers and Associate Editors, a reason of great concern for us; because English language is often so unsatisfactory, and in some cases it prevents content analysis.

I request the Authors to collaborate with the BJCVS by sending us the manuscripts reviewed by professionals who are able to translate them accurately, thus, they won't be returned, there will be no delays or higher costs to be paid by the authors. Our reviewers do not correct articles, they only check if the English language is good enough to be published.

We would like to ask you to read the editorial written by Dr. Mariel Marlow^[1]^, published in Clinics, which brings together the 10 most common mistakes made by Brazilians when writing English manuscripts.

## SOCIAL MEDIA

Under the guidance of the BJCVS team, the Junior Editor Dr. Gabriel Liguori took over the social media two months ago and created a team composed of Residents in Cardiovascular Surgery and Medical Academics, who update the Blog https://bjcvs.wordpress.com and the BJCVS Facebook page https://www.facebook.com/bjcvs.

### Facebook

**Fig. 1:**
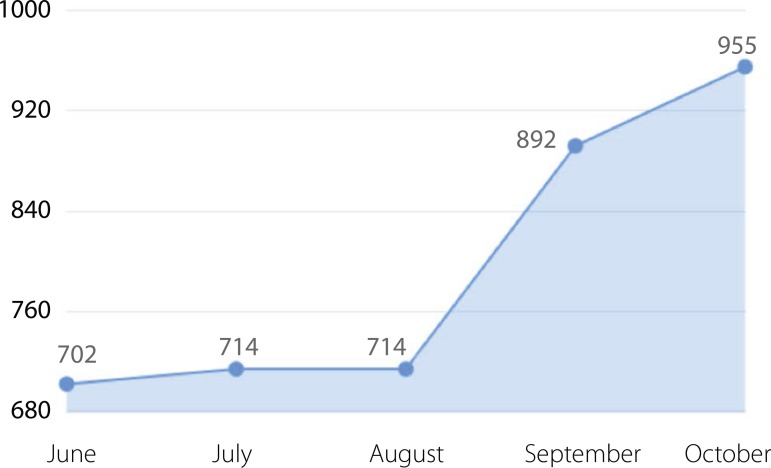
Likes of the BJCVS Facebook Page.

**Fig. 2:**
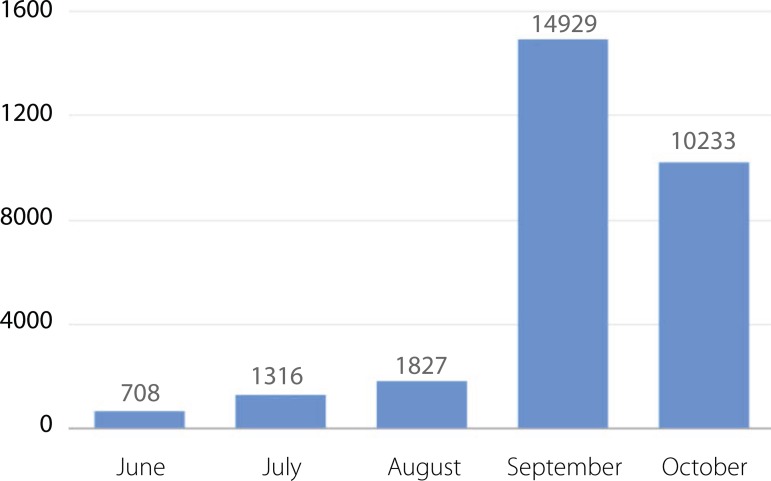
Number of people interacting with the BJCVS Facebook Page.

After posting the material, the number of likes in 2 months (Figure 1) was increased by 33%, there was also an eight-fold increase in the number of people interacting with us in the first month (Figure 2)

### Blog BJCVS

**Fig. 3:**
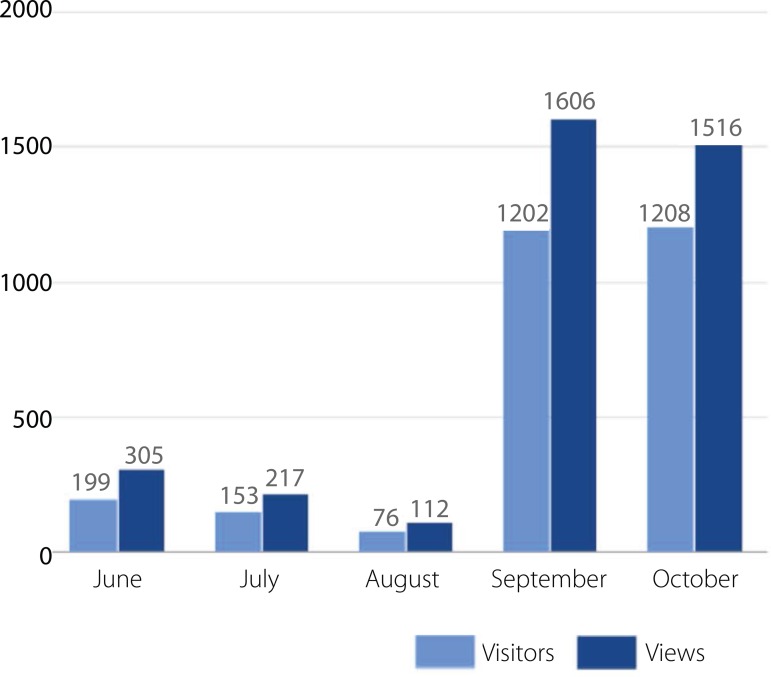
Number of visitors and views of the BJCVS blog.

We had more than a 15-fold increase in the number of visitors and more than a 14-fold increase in the number of blog views in the first month (Figure 3).

Top Posts by Blog Viewing:

1. Forming a Cardiac Surgeon - The New American Alternatives - 1,103 views
2. How a Cardiac Surgeon Won a Nobel Prize in Medicine: The Invention of Catheterization - 550 views
3. The woman as a cardiovascular surgeon: Is the cup half empty or half full? - 516 views

### BJCVS website

**Fig. 4:**
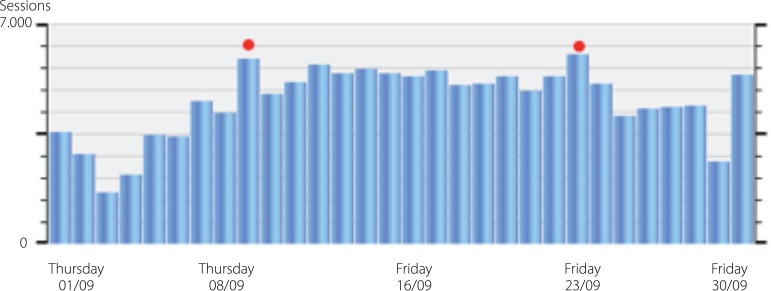
Accesses in September.

**Fig. 5:**
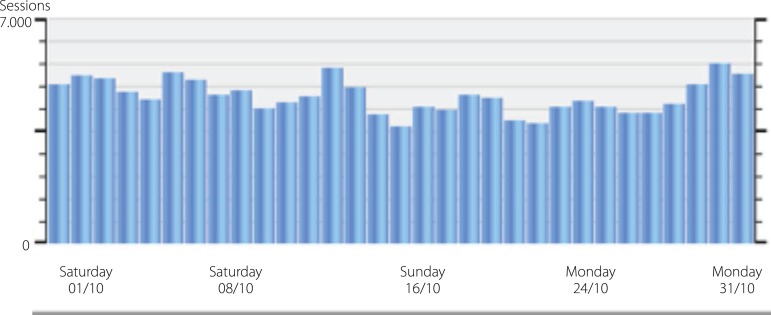
Accesses in October.

Access to BJCVS pages in September and October 2016 (Figures 4 and 5):

These accesses correspond only to those related to our site. We have a similar number of readers accessing our journal through Scielo or other Databases.

## ARTICLES AND CME

In this issue, there are two international editorials and 10 articles, eight of them are national and two international in the original article, review article and how-to article categories.

The following articles that have been chosen to be part of Continuing Medical Education (CME) are also available, now under the responsibility of an Associate Editor specific to this important area: "*In-Vitro* Evaluation of Two Types of Neonatal Oxygenators in Handling Gaseous Microemboli and Maintaining Optimal Hemodynamic Stability During Cardiopulmonary Bypass" (page 343); "Intraoperative Analysis of Flow Dynamics in Arteriovenous Composite Y Grafts" (page 351); "Comparison of Early Outcomes with Three Approaches for Combined Coronary Revascularization and Carotid Endarterectomy" (page 365) and "Reference Values for the Six-Minute Walk Test in Healthy Children and Adolescents: a Systematic Review" (page 381).

I emphasize that the CME is a valuable tool for learning and updating the knowledge, besides being important for the test to obtain the Specialist Title of the SBCCV.

As always, I deeply thank all the Directors of the SBCCV, Associate Editors, Peer Reviewers, all the dedicated staff that help me a lot, and, finally, but not less important, I also thank the Authors who give prestige to our Journal sending the manuscripts to be considered for publication.

Sincerely,

Domingo M. Braile

^1^Editor-in-Chief - BJCVS
